# Fusion-Based Body-Worn IoT Sensor Platform for Gesture Recognition of Autism Spectrum Disorder Children

**DOI:** 10.3390/s23031672

**Published:** 2023-02-03

**Authors:** Farman Ullah, Najah Abed AbuAli, Asad Ullah, Rehmat Ullah, Uzma Abid Siddiqui, Afsah Abid Siddiqui

**Affiliations:** 1 Division of Computer Science and Engineering, Jeonbuk National University, Jeonju 54896, Republic of Korea; 2 College of Information Technology, United Arab Emirates University (UAEU), Abu Dhabi 15551, United Arab Emirates; 3 Department of Electrical and Computer Engineering, COMSATS University Islamabad-Attock Campus, Attock 43600, Pakistan; 4 Department of Computer Systems Engineering, University of Engineering and Technology, Peshawar 25000, Pakistan

**Keywords:** autism spectrum disorder, sign language, gesture recognition, speech-impaired, IoT, artificial neural network, decision tree, random forest, k-nearest neighbors

## Abstract

The last decade’s developments in sensor technologies and artificial intelligence applications have received extensive attention for daily life activity recognition. Autism spectrum disorder (ASD) in children is a neurological development disorder that causes significant impairments in social interaction, communication, and sensory action deficiency. Children with ASD have deficits in memory, emotion, cognition, and social skills. ASD affects children’s communication skills and speaking abilities. ASD children have restricted interests and repetitive behavior. They can communicate in sign language but have difficulties communicating with others as not everyone knows sign language. This paper proposes a body-worn multi-sensor-based Internet of Things (IoT) platform using machine learning to recognize the complex sign language of speech-impaired children. Optimal sensor location is essential in extracting the features, as variations in placement result in an interpretation of recognition accuracy. We acquire the time-series data of sensors, extract various time-domain and frequency-domain features, and evaluate different classifiers for recognizing ASD children’s gestures. We compare in terms of accuracy the decision tree (DT), random forest, artificial neural network (ANN), and k-nearest neighbour (KNN) classifiers to recognize ASD children’s gestures, and the results showed more than 96% recognition accuracy.

## 1. Introduction

Autism spectrum disorder (ASD) is a complex neurological developmental disorder characterized by significant impairment in social interaction, communication, and ritualistic behavior [1]. According to the 2015 World Health Organization statistics, more than 5% of the world’s population suffers from hearing impairment. The burden on society increases due to deaf workers because their unemployment rate is about 75% [2]. About 1 out of 200 children is diagnosed with ASD, and boys are four to five times more affected than girls [3]. Biao et al. [4] stated that according to the Centers for Disease Control and Prevention (CDC), about 1 in 59 children has ASD. Maenner et al. [5] discussed in the recent CDC estimates that in eight-year-old children, 23 out of 1000 (i.e., 1 in 44) meet the criterion of ASD, which is an increase from the prior estimates. Areeb et al. [6] reported in their article that according to the 2011 census, in India, about 2.7 million people cannot speak, and 1.8 million are deaf. Each ASD child has his/her specific needs. ASD symptoms are usually seen in a child aged one to two years. ASD children often experience problems with social contact, have an association, and lack social interaction, social behavior, or physical activity. ASD children have difficulties communicating with other people. These children require extra care because they often do not understand other people’s actions. These children are challenging to understand and face difficulty in speech and communication. Ramos-Cabo et al. [7] studied the impact of gesture types and cognition in ASD. In this paper, we propose a hand- and head-worn sensors-based system to recognize the sign language of autistic children to communicate the message to non-ASD people.

Autistic people can communicate in sign language, which is not easy to understand [8,9] because only deaf and mute people understand sign language. It is difficult for people to learn sign language until it becomes necessary, or they must encounter ASD people regularly [10]. Padden, in the article [11], identified that there are 6909 spoken languages and 138 sign languages. Sign language focuses on the arms, hands, head, and body movements to construct a significantly gesture-based language. Providing the proper facilitation to disabled people is essential because a considerable portion of our society consists of autistic hearing-impaired people. Traditional and non-technical solutions offered to these people are cochlear implants, writing, and interpreters. A cochlear implant is not an inclusive solution, as 10.1% of people having cochlear implants still have hearing problems [12]. It is frustrating to use writing as a mode of communication because of its ambiguity and slowness. A sensor added to the body requires tight-fitting garments that upset the solace level to avoid relative advancement. In contrast, handwriting rates are 15 to 25 WPM (words per minute), usually a person’s second language. Autistic people also use interpreters, but this involves privacy issues, and they charge a high hourly rate. Finding an interpreter for a person with specialized vocabulary is also challenging. Ivani et al. [13] designed and implemented an algorithm to automatically recognize small and similar gestures within a humanoid–robot therapy called IOGIOCO for ASD children. Wearable computing provides proactive support in collecting data from the user context. Wrist-worn sensor modules are used for collecting the gestures data and recognizing gestures of the ASD children [14]. Borkar et al. [15] designed a glove of flex sensors pads to sense various movements, specifically the curve movements of fingers. The device is designed smartly to sense the resistance and action by the hand. Most of the literature is focused on hand movements only and is limited in recognizing complex gestures which involve multiple kinds of body movement. In this article, we propose a fusion-based IoT platform where multiple sensors are placed on different body locations for complex gesture recognition of ASD children.

Wearable sensors, the Internet of Things (IoT), machine learning, and deep learning have gained a significant role in daily life. IoT integrates the actuators, sensors, and communication technologies to acquire the data, control the environment, and access statuses at any time and anywhere [16,17,18]. Features are extracted from sensor data and then classified by different classification techniques. Placing the sensors at an optimal location is essential, as the variation affects the classification. Avoiding relative movement sensors appended to the body requires the utilization of tight-fitting garments which upsets the solace level [19]. A gesture-based recognition system is the best possible solution which enables mute people to move quickly through society. A sensor-based recognition system has advantages over other recognition approaches. The benefits are that the sensors are portable, easy to use, low power, low cost, durable and safe to use, and easy to install [20]. Some of the gestures of these people are complex, and they require a particular interpreter, so there is a need for a system that efficiently translates the gestures of mute and deaf people.

The overall contributions of the paper can be summarized as follows:

- ASD children have difficulties in communication skills and they suffer from a speech disorder. We proposed a multi-sensor body-worn area sensor network for complex sign language recognition of ASD children focusing on helping the ASD children to convey their message to non-ASD people.
- We acquired and built a dataset of ten complex gestures of ASD children’s sign language, performed twenty times each using the designed flex sensors glove, accelerometers, and gyroscope placed at hand and head position.
- We extracted statistical measures as features from the sensors data both in time and frequency domains to reduce the processing time and improve accuracy.
- A performance comparison of machine learning algorithms to select the algorithm with the highest accuracy, precision, and recall for efficient recognition of ASD gestures was carried out.
- A real-time implementation is tested on Raspberry-PI systems on a chip to demonstrate the system.

The rest of the paper is organized as follows. Section 2 briefly introduces the related works and the background of sensors, IoT, and machine learning techniques about activities and gesture recognition. The proposed methodology for body-worn sensors-based IoT platform for ASD children’s gestures acquisition, feature extraction, and recognition algorithms is discussed in Section 3. Section 4 discusses the results, and finally we conclude the research in Section 5.

## 2. Related Work

Body area sensor network (BASN), also termed wireless body area network (WBAN), is a network for portable processing devices. BASN devices can be incorporated into the body as an implant, attached to a body at a fixed position or in combination with devices that people can carry in multiple positions, such as pockets, in the hand, or in carrying bags [21]. There is always a trend toward narrowing devices; body area networks consist of multiple mini sensor networks, and collectively these sensor networks are called body central units (BCUs). Larger smart devices (tabs and tablets) and associated devices play a significant role in serving as data centers or data portals, and provide a user interface to view and manage BASN applications on-site [22]. WBAN can use WPAN (wireless personal area network) technology as long-distance installation [23]. IoT makes it possible to connect portable devices implanted in the human body to the internet. In this way, healthcare professionals can access a patient’s data online, using the internet, regardless of the patient’s location. TEMPO 3.1 (tech-medical sensitive observation) allows wireless transmission in six degrees, in a portable form, to capture and processes the precise and accurate platform of third-generation body area sensors. TEMPO 3.1 is designed for user and researcher use, allowing motion capture applications in BASN networks [24].

Human gesture recognition uses ambient sensors, cameras, wearable sensors, and mobile sensor-based systems. Ambient sensors, used in an external or local frameworks, are installed in the environment and have no physical contact with the concerned person. These sensors include radar, RF signals, event (switch based), and pressure sensors [25,26]. The systems have the main advantage of allowing ignoring the existence of the sensors within the user environment, and not pertaining to privacy leakage issues. For example, to monitor the older person’s activity, sensors can be installed in a living room or anywhere in the home [27]. Cameras also achieve gesture detection, in addition to ambient sensors. The cameras are placed in a limited area to provide images or videos of human activities to implement the fall detection algorithm [28]. Zheng et al. [29] proposed a large-vocabulary sign language recognition system using an entropy-based forward and backward matching algorithm to segment each gesture signal. They designed a gesture recognizer consisting of generating a gesture and a semantic-based voter. The candidate gesture generator aims to provide candidate gesture designs based on a three-branch convolutional neural network (CNN). Han et al. [30] proposed three-dimensional CNN for concurrently modeling the spatial appearance and temporal evolution for sign language recognition. They used RGB video to recognize the signs. To reduce risks and improve the quality of life at home, it can be monitored by determining daily life activities (ADL) using RGB-D cameras [31]. Camera surveillance does not attract many people as it raises privacy concerns in general. The camera is especially suitable for living rooms; however, it is difficult to place a camera in a living room due to privacy. Recently, electronic devices (such as smartphones) are becoming a daily de facto tool. Mobiles have many integrated sensors such as GPS locators, gyroscopes, and accelerometers [32,33]. These sensors provide data both remotely and accurately. In addition to many advantages, reception based on portable sensors also has disadvantages. Keeping the smartphone in pockets reduces the effectiveness of recognizing certain activities such as writing, eating, and cooking [34]. In addition, women tend to keep their phones in their bags, almost losing mobile connection to their bodies [35,36]. We can eliminate all these problems and achieve greater efficiencies by installing wearable sensors on desired body parts.

Wearable devices give opportunities for innovative services in health sciences, along with predictive health monitoring by persistently collecting data of the wearer [37]. Wearable sensors provide precise and accurate data by installing them on the desired muscles of the limb, thereby ensuring a better and more correct method of gesture recognition system [38]. This system comprises body-worn sensors by the person for data acquisition purposes. The systems contain body-worn accelerometers, gyroscopes, flex sensors, pedometers, and goniometers [39]. The sensors are installed at different body parts, such as the hip, wrist, ankle, arm, or thigh, to recognize gestures performed by these muscles [40]. The advantage of using wearable sensors is that data collected by these sensors have greater efficiency, can monitor multiple muscles’ movement, and above all, are effortless to use for the affected person [41]. The study on gesture recognition has found that the location of sensors depends primarily on the purpose of data collection. The accuracy of the observation depends on the position of the sensor installed on the body [42]. It also shows that different gestures, which include movement, posture, or activity, are best controlled by placing a sensor on the ankle, hip, or pocket [43]. On the other hand, exercises related to upper body parts require sensors placed on the chest, neck, arms, or elbows for better recognition accuracy [44]. The system proposes the optimal location for the sensor network to be placed on different muscles to provide better accuracy. Li Lang et al. [45] designed SkinGest using five strain sensors and machine learning algorithms such as k-nearest neighbor (KNN) and support vector machine (SVM) to recognize the numerical sign language for 0–9. They used ten subjects to acquire data. Zhang et al. designed WearSign for sign language translation using the inertial and ElectroMyoGraphy sensors [46]. Table 1 shows the literature review related to wearable sensors.

The sensor data is collected using different sampling frequencies depending on the nature of the recognizing activities. The data is divided into segments of time series, called window size. Zappi et al. [47] performed several related tasks and collected data from an accelerometer with a frequency of 50 Hz and proposed a two-step selection and acquisition method. The acquisition phase received 37 functions with a one-second window size and overlapping of half a second. They used the relief-F function for the selection algorithm to select 7 attributes specific to 37 in the selection step [48]. First, they used a low-pass filter to pre-process the signal to eliminate the DC component, then recover the processed data to gain the desired properties (min, max, average RMS value, STD, average, and maximum frequency). Classifiers are one of the best methods that use functional data for testing and training. Training data generally include functions that are not labeled. The classifier learning algorithm adjusts the parameters to create a model or run a hypothesis. Now this template can provide a label to new inputs [49]. The most used classifiers techniques used for the classification of data are random forest (RF), KNN, SVM, multilayer receiver perception (MLP), artificial natural network (ANN), and decision tree. Table 2 shows the literature review related to different algorithms.

## 3. The Proposed Body-Worn Sensors-Based IoT Platform for ASD Children Gesture Recognition

**Figure 1:**
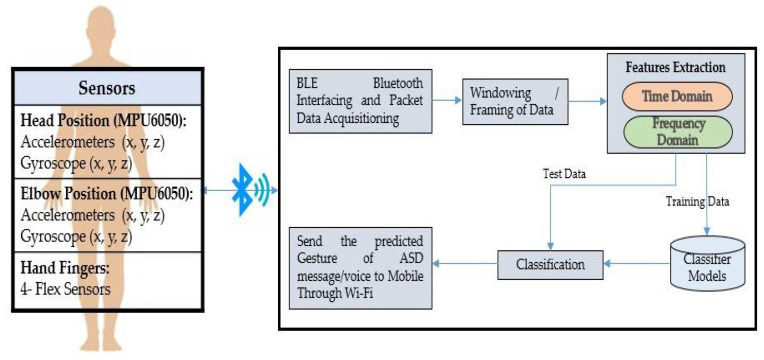
The proposed architecture for body-worn sensors-based IoT platform for ASD children’s gestures recognition.

The article aims to develop a body-worn multi-sensor-based IoT platform to recognize ASD children and convert sign language to voice and text messages. Figure 1 shows the system architecture proposed for the sign language translator that explains the complete system operation. The proposed system consists of the following modules:

- Body-worn sensors interfacing platform
- Pictorial overview of gestures and data acquisition
- Sampling, windowing, and features extraction
- Classification/recognition algorithms

### 3.1. Body-Worn Sensors Interfacing Platform

**Figure 2:**
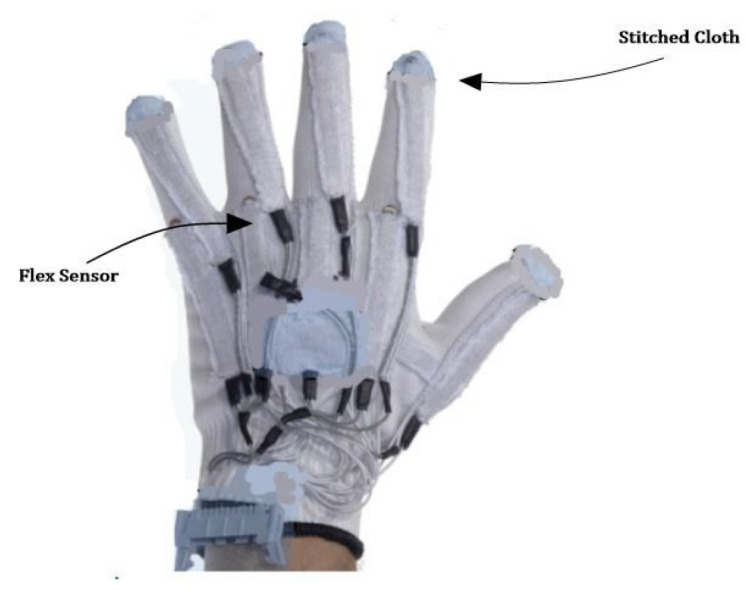
Placement of flex sensors in glove for hand gesture data.

We use body-worn sensors placed in three positions. (1) At the head position, in a head cap, an MPU6050 sensor module is installed. (2) At an elbow position, an MPU6050 sensors module is installed. For the fingers movements and bending, we use Flex sensors. An MPU6050 sensors module consists of an accelerometer and gyroscope of 3-axis. For the Flex sensors, we designed a glove, as shown in Figure 2, to place the Flex sensors in order to acquire finger-bending movement. The flex sensors can be used as variable resistors, and their resistance is proportional to the bend in the fingers. When the fingers bend, the resistance increases and vice versa. The flex sensor has flat resistance of 25 kΩ. Depending on a bend, the value can increase up to 125 kΩ. Fixed resistors uses flex sensors in series. Equation (1) shows the voltage divider rule, which calculates the flex resistance.

(1) $$\begin{array}{c} V_{out}=V_{in}\left(\frac{R_{1}}{R_{1}+R_{2}}\right) \end{array}$$

where $V_{out}$ = voltage of flex sensor, $V_{in}$ = 5 V (in this case); $R_{1}$ = resistance of flex sensor and $R_{2}$ = fixed resistance. MPU6050 is the motion sensor used in this paper to measure the linear and angular motion of the head and hand. This module has a three-axis embedded accelerometer and three-axis gyroscope sensors. The accelerometer and gyroscope of the MPU6050 both provide continuous output over time. For sending the data to the controller using the I2C communication protocol, the values are digitized using a 16-bit analog/digital converter. The ADC samples the data with a specific frequency (fs) and then quantizes these samples. We used the sampling frequency of 50 Hz for the accelerometer and gyroscope. The data from the sensors is collected by Arduino, which is then sent to the Raspberry Pi using Bluetooth. Data from the motion sensor is compiled using the I2C protocol, and data from the flex sensor is collected at the analog input (ADC). I2C is a two-wired bidirectional serial communication protocol used to send and receive data between two more electronic circuits.

### 3.2. Pictorial Overview of Gestures and Data Acquisition

**Figure 3:**
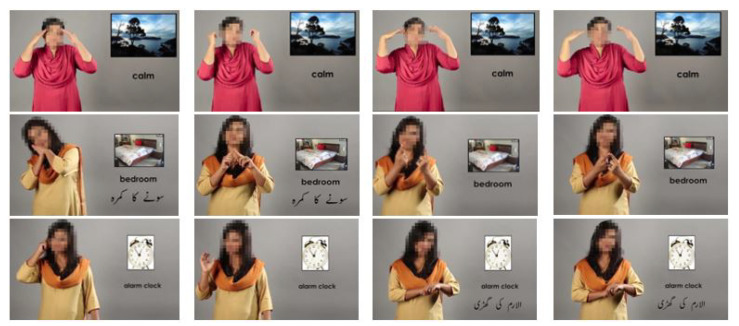
Pictorial overview of different gestures performed.

**Figure 4:**
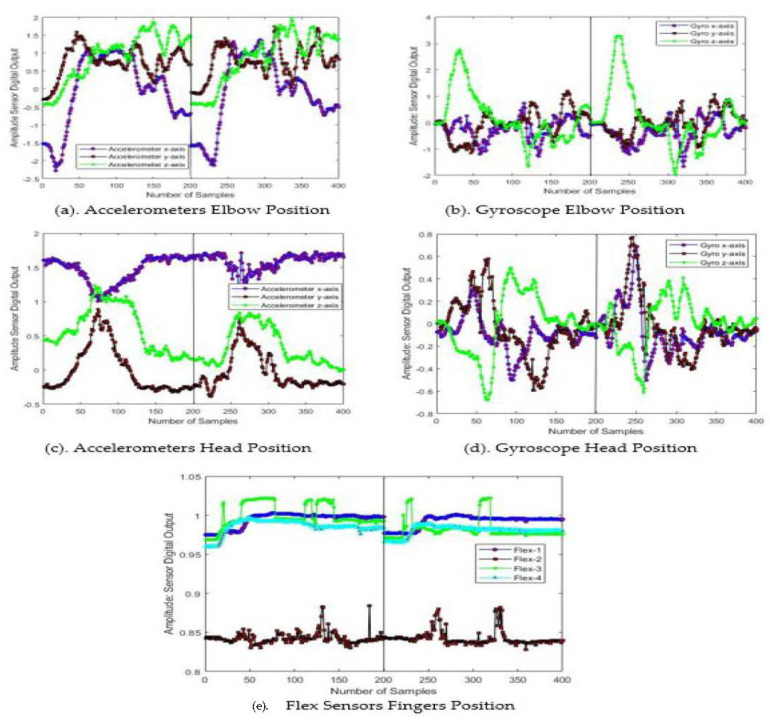
Two cycles of sensor response for acquiring the gesture alarm clock.

In this article, we acquire the data of ASD children by installing motion sensors at elbow and head positions, and a flex sensors glove for finger bending. Figure 3 shows the pictorial overview images taken from Pakistan sign language (PSL) and clearly depicts that each gesture involves multiple parts of the body. We installed multiple sensors on different body parts. We collected the data for ten gestures from ten ASD children from MPU1, MPU2, and flex1 through flex4, and assigned labels as GST-1–GST-10 as shown in Table 3. Each ASD child performed each gesture 20 times. Figure 4 shows the sensors’ response for two cycles performed by an ASD subject for the gesture of GST-1: alarm clock. We consider the 200 samples in a cycle, i.e., 4 second to complete each gesture. The Figure 4 clearly depicts that the sensors’ responses have a similar nature with little variation in amplitude and time.

### 3.3. Sampling, Windowing, and Features Extraction

The sampling rate and windowing for feature extraction play a leading role in the gesture recognition process. The accelerometer, gyroscope, and flex sensor data is acquired at 50 Hz. Four seconds for the non-overlapping sliding windows approach is used for features . The features are extracted using the data window of 200 samples. We extract the features such as standard deviation, mean, minimum, and maximum values of accelerometers and flex sensors. The RMS feature is only used for the gyroscope, and the remaining features are only for accelerometers. Table 4 shows the statistical measures used to extract the features for classifying and recognizing ASD children’s gestures. We use a feature vector of 58 for each gesture.

### 3.4. Classification and Recognition Using Machine Learning Algorithms

Raspberry-PI is used as a platform to extract the features and use the machine learning libraries to recognize the gesture. We used the KNN, decision trees, random forest, and neural networks. We explain the algorithms in the following subsections.

#### K-Nearest Neighbours (KNN) Algorithm

The KNN algorithm is one of the lazy methods used for learning, because the learning (discovering the relationship between input features and the corresponding labels) begins after a test input. The algorithm finds the similarities (distance) between the feature vectors, sorts according to the similarity measure, and selects the Top-K neighbors. From the training data, this algorithm finds the K number of adjacent samples that are like test input samples. Mathematically, the similarity between the training samples and test data can be calculated either by Euclidean, Manhattan, or Minkowski distance, as follows, by Equations (14)–(16), respectively. Algorithm 1 shows the

(14) $$D=\sqrt{\sum_{i=1}^{n}{\left(x_{i}-y_{r_{i}}\right)}^{2}}$$

(15) $$D=\sum_{i=1}^{n}\left|x_{i}-y_{r_{i}}\right|$$

(16) $$D={\left(\sum_{i=1}^{n}{\left|x_{i}-y_{r_{i}}\right|}^{p}\right)}^{\frac{1}{p}}$$

**Algorithm 1** KNN Pseudocode
1:Assign (**Tr**, **Cl**, **z**)2:**Tr:** training data, **Cl:** classes labels of **T**, **z:** unknown sample3: 4:**for***j* = 1 to *n* **do**5:    Find the distance: D(Tr(j), z) using either of Equations (14)–(16)6:
**end for**
7:Calculate the set K, which consists of the indexes for the lowest distance D($T_{j}$, z)8:Return majority label for $Cl_{K}$


### 3.5. Decision Tree

The fundamental structure of a decision tree includes a root node, a branch, and a leaf node. The root node represents an attribute’s test, the branches give the test outcomes/results, the leaf node gives the decision taken after considering the attributes (in other words, leaf nodes give a class label), and internal sub-nodes signify the dataset structures. It creates a tree type for the whole dataset and proceeds a single outcome at each leaf node by minimizing errors. Two parameters play an important key role in the formation of this algorithm’s structure: the attributes and attributes selection method. The nodes are selected using various mechanism scuh as Gini-Index, entropy, and information gain ratio. The pseudo-code of decision tree is Algorithm 2.
**Algorithm 2** Decision Tree Pseudo-code1:Create a node $N_{d}$2:**if** tuples in *X* belongs to same class, Cls **then**3:    return $N_{d}$ in the form of leaf node labeled as class Cls4:**else if**attributelist is empty **then**5:    return $N_{d}$ as leaf node labeled as majority class in *X* // based on majority voting6:**end if**7:Apply attribute-selection-method (*X*, attribute list) to find the “best” splitting criterion; label node $N_{d}$ with splitting criterion8:Let $X_{i}$ be the set of data tuples in *X* satisfying outcome *i* // a partition9:**for** each outcome *i* of splitting criterion10:// partitioning of data and growing of sub-trees related to each partition **do**11:    **if** $X_{i}$ is empty **then**12:        Attaches a leaf labeled with the majority class in *X* to node $N_{d}$13:    **else**14:        Attach the node returned by generate-decision-tree ($X_{i}$, attribute list) to node $N_{d}$15:    **end if**16:**end for**17:Return $N_{d}$

#### 3.5.1. Random Forest Algorithms

The random forest method [60] is a classification method used to build multiple decision trees and ultimately take many weak learners’ decisions. Often, pruning these trees helps to prevent overfitting. Pruning serves as a trade-off between complexity and accuracy. No pruning implies high complexity, high use of time, and the use of more resources. This classifier helps to predict the gesture. Algorithm 3 shows the pseudo-code of random forest.
**Algorithm 3** Random Forest Pseudocode1:Randomly choose f features from n features f < n,2:Calculate the node d using the finest split-point,3:Divide nodes into child nodes using the best division point,4:Repeat above steps from 1 to 3 until the number of nodes *I* has reached,5:Build a random forest by repeating the above all steps *N* times to create *t* number of trees.

#### 3.5.2. Artificial Neural Network

This paper uses neural networks [61] for complex models and multi-class classification. Neural networks are inspired by the brain, which is a network of neurons. The neuron model consists of input with input weight (activation function), hidden layers, and output. When input arrives, it is multiplied by the weight of the connection. During the time of the training of the model, this weight is updated to reduce the error. The input layer of the model does not process the input and passes it on to the nest layer, called hidden layers. These layers process the signals and create an output delivered to the output layer. The weight of the connection defines the influence of one neuron over the other. This weight updates in the back-propagation process to reduce the error. Algorithm 4 shows the neural networks.
**Algorithm 4** The Neural Networks using Back-propagation Method Pseudocode1:Initialize the weights and learning rate *n* with the appropriate values2:**Input:** Enter the training data3:**Output:** Obtain the output of the network4:**for** Calculate the error **do**
(17)E=Y−y
(18)$$\Delta =\phi^{{}^{\prime }}\left(w\right)E$$
where5:$\phi^{\prime }$ is the derivative of the leaf (output) node activation function,6:*y* is the output from the leaf node,7:*Y* is the actual output, and8:*w* is the weight of the node.9:Procreate the leaf node backward, and calculate the deltas of the next nodes
(19)$$E^{\left(n\right)}=W^{T}\delta$$
(20)$\Delta^{\left(n\right)}=\phi^{{}^{\prime }}\left(w^{\left(n\right)}\right)E^{\left(n\right)}$10:**end for**11:The above loop continuously runs until the hidden layer reaches the right next to the input layer,12:Weights adjustment will be carried out according to the learning rule as given below
(21)$$\Delta z_{kl}=\alpha \delta_{k}x_{l}$$
(22)$z_{kl}=z_{kl}+\Delta z_{kl}$13:**Repeat** above 2 to 7 steps for each point.

After the gesture is recognized, we send the gesture text message to the person/guardian’s mobile for interaction.

## 4. Results and Discussion

This section discusses the sensors’ response and the results of classifiers. The inputs to these classifiers are the features extracted from the original dataset. We used accuracy, precision, and recall as a performance metrics to evaluate and compare the algorithms.

### 4.1. K-Nearest Neighbours (KNN) Algorithm Results

**Figure 5:**
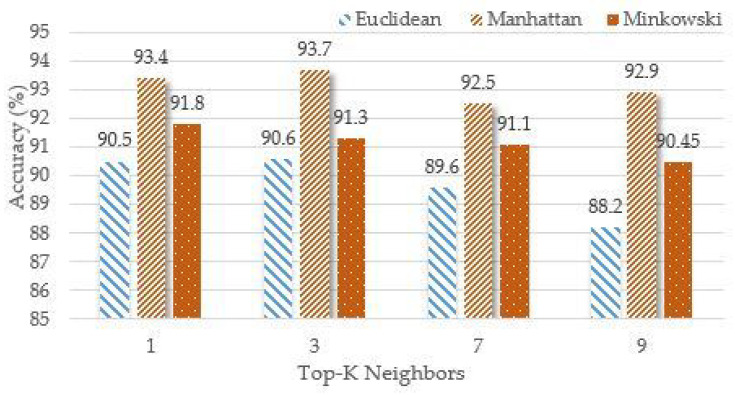
The KNN algorithm accuracy using different K-values and distance measures.

This classifier has several distances to use. Here, we have used three different distances: Euclidean distance, Manhattan distance, and Minkowski distance. The value of K varied between 1, 3, 7 and 9 to obtain maximum accuracy. The KNN classifier is applied to the dataset with different K values and the distance measures as shown in Figure 5. The maximum accuracy achieved with the KNN algorithm is 93.7% using Manhattan distance at K = 3 and cross-validation of 10 folds. Table 5 shows the confusion matrix for maximum accuracy of KNN.

### 4.2. Decision Trees (J48) Results

The results from J48 classifiers are summarized in Table 6. We used different cross-validations to check the effect of training the dataset size for accuracy. The maximum accuracy achieved is for 90–10% for ten-fold cross-validation.

The maximum accuracy obtained using this classifier is 87.912% at a cross-validation of 10%. Table 7 shows the corresponding confusion matrix.

### 4.3. Random Forest Results

The results in Table 8 show the output with changing cross-validation and maximum depth.

The maximum accuracy obtained is 95.9%. Table 9 shows the resulting confusion matrix of this maximum accuracy.

### 4.4. Artificial Neural Network

**Figure 6:**
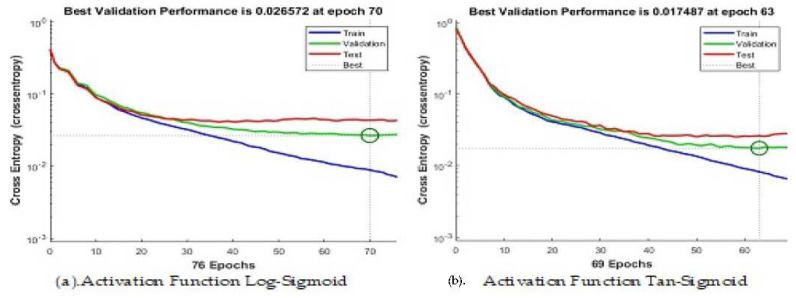
The error convergence and optimization current using different activation functions and neurons = 100.

**Figure 7:**
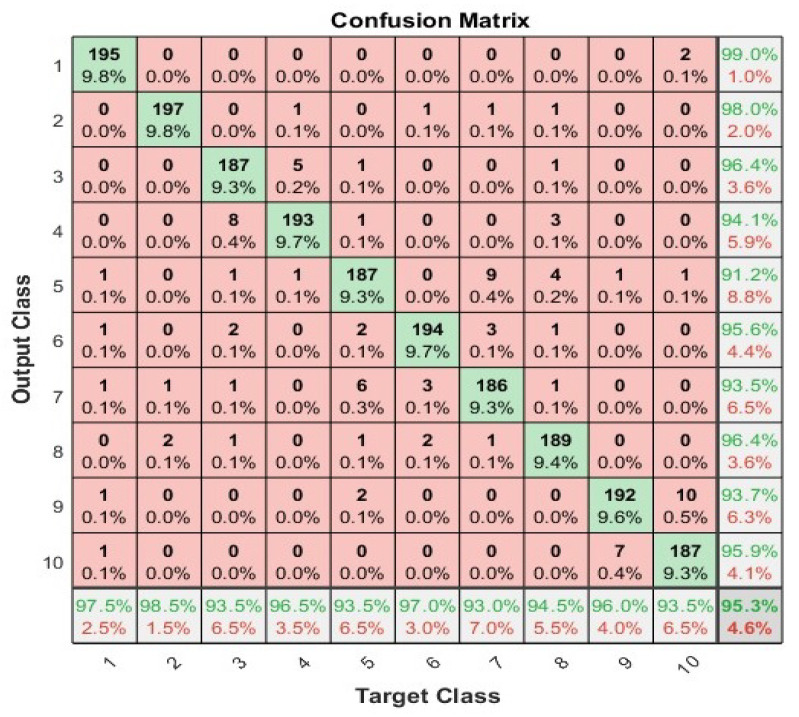
Confusion matrix of neural networks using activation function log-sigmoid.

**Figure 8:**
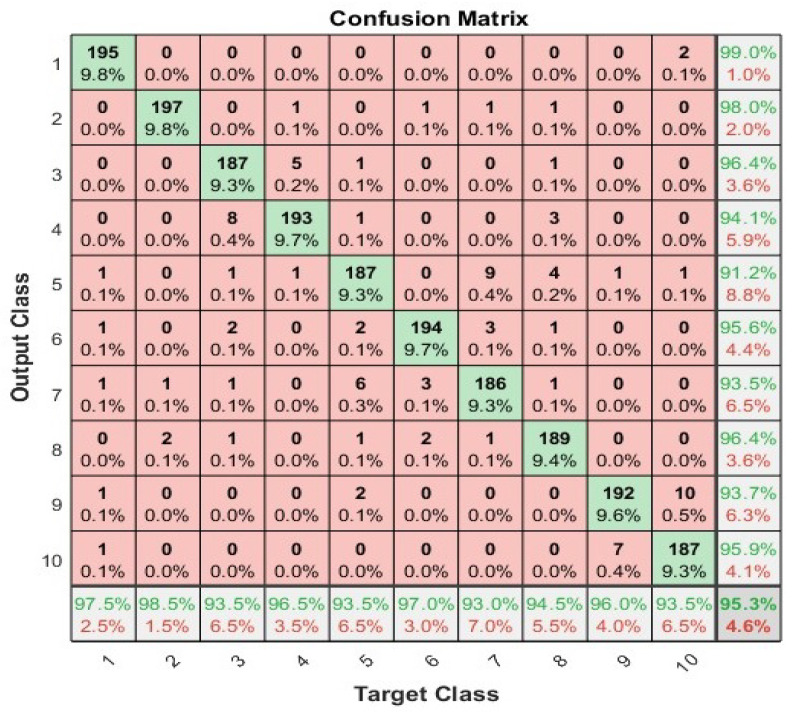
Confusion matrix of neural networks using activation function tan-sigmoid.

We have applied the neural network with 70%, 15%, and 15% split for training, validation, and testing, respectively, to show the highest accuracy. We used 100 neurons in the hidden layer and two different activation functions i.e., log-sigmoid and tan-sigmiod. Figure 6 shows the convergence curve using cross entropy for both activation functions. The tan-sigmoid shows early convergence with higher accuracy. Figure 7 and Figure 8 show the confusion matrices of log-sigmoid and tan-sigmoid, respectively.

### 4.5. Comparison of Classifiers

**Figure 9:**
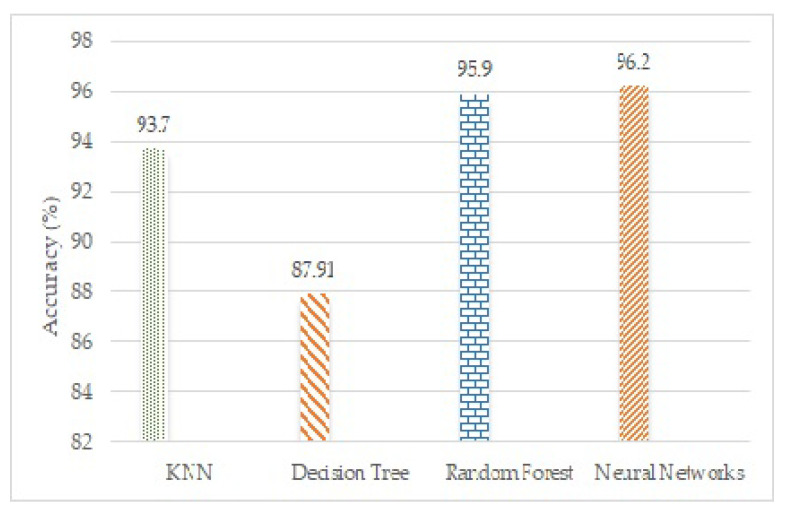
Performance Comparison Graph.

Figure 9 shows a bar graph for the comparison of different classifiers. The bars represent the average accuracy of each classifier. The neural networks classifier with tan-sigmoid has the highest average accuracy of all.

## 5. Conclusions

This article proposed a wearable sensors-based body area IoT system to acquire ASD children’s gesture time-series data and use machine learning (ML) to recognize what they are trying to say in sign language. This research is focused on the daily gesture recognition of ASD children to communicate their message to non-ASD people without any hesitation. The proposed system consists of wearable sensors such as accelerometers, gyroscopes, and flex sensors modules, installed at the head, elbow, and hand–fingers positions. The proposed system consists of acquiring the time-series data, sending the data through BLE Bluetooth to a Raspberry-PI-based processing system to extract the features, and recognizing the gesture using ML algorithms. Complex gestures involve the movement of multiple body parts, so we use multiple sensors at the head, hand, and finger to acquire the motions. We use two motion sensor modules (one for the head and one for the hand at the elbow) and four flex sensors (one for each finger) to collect the gesture response. We collected data from sensors and extracted features of 200 samples for every gesture performed. We have used different classifiers such as k-nearest neighbor, random forest, decision tree, and neural networks to predict the gesture performed. On average, the accuracy obtained is more than 90% for each gesture, and the maximum accuracy achieved is 96% by the neural networks. Finally, the gesture predicted is displayed for the gesture to verbal communication through the WiFi on the user’s smartphone.

## Figures and Tables

**Table 1:** Literature review focused on wearable sensors used for gestures and activity recognition.

Ref. No.	Dataset Details	Extracted Features	Types of Sensor
[50]	20 numbers, 20 alphabets	Mean value, standard dev, percentiles. and correlation frequency domain (energy, entropy)	5 capacitive touch sensors
[43]	200 words, 10 iterations	Mean, standard dev, kurtosis, skewness, correlation, range, spectral energy, peak frequencies, cross-spectral densities, and spectral entropy	2 accelerometers
[51]	35 gestures, 20 trials each 5 persons	Mean value, variance, energy, spectral, entropy, and FFT	2 accelerometers, 1 gyroscope
[52]	Arabic alphabets, 20 trails	Average, standard dev, the time between peaks, binned distribution, and average resultant acceleration	2 accelerometers, 2 gyroscopes
[53]	50 letters, 50 iterations	Mean, median, standard deviation, frequency domain, time domain	5 flex sensors
[49]	20 gestures, 4 persons, 20 iterations	Mean, variance, energy, spectral entropy, and discrete FFT	2 accelerometers, 1 gyroscope
[8]	English alphabets	Mean, standard deviation, average	1 accelerometer

**Table 2:** Literature review related to algorithms

Ref. No.	Paper Proposal	Hardware/Software	Datasets	Applied Algorithms	Accuracy
[54]	This paper proposes an electronic system equipped with wearable sensors for gesture recognition to interpret abnormal activities of the children to their parents through a machine learning algorithm.	Flex Sensor, Arduino, mpu6050, Bluetooth	Alphabets, 20 iterations	KNN	95%
[55]	This work provides ICT solutions for autistic children by examining a person’s voice, body language, and facial expressions to monitor their behavior while performing gestures.	Accelerometer, PC, RGB camera	40 gestures, 10 persons, 10 repetitions	Parallel HMM	99%
[56]	This article presents the links between known attention processes and descriptive indicators, emotional and traditional gestures, and nonverbal gestures between ASDs in attention processes and gestures.	Flex sensor, MPU 6050, contact sensor, Arduino, Bluetooth, PC	1300 words	HMM	80%
[57]	This paper has proposed a system in which the recognition process uses a wearable glass and allows these children to interpret their gestures easily.	RGB camera, PC	26 postures, 28,000 images	HAAR cascade algorithm	94.5%
[58]	This paper presented the idea to analyze the development of conventional gestures in different types of children, such as typical ASD children.	RGB camera, PC	English and Arabic alphabets	BLOB, MRB, least difference, GMM	89.5%, 80%
[59]	This paper presented the IoT-based system called” Wear Sense” to detect the atypical and unusual movements and behaviors in children who have ASD.	Capacitive touch sensor, R-pi, Python	36 gestures, 30 trials each	Binary detection system	86%

**Table 3:** Information about the gestures.

Gesture	Label	Gesture	Label
Alarm clock	GST-1	Brilliant	GST-6
Beautiful	GST-2	Calm	GST-7
Bed	GST-3	Skull	GST-8
Bedroom	GST-4	Door Open	GST-9
Blackboard	GST-5	Fan	GST-10

**Table 4:** Feature extraction from time-series data of multiple sensors

Features Name	Description	Equations
Mean	Mean value calculation for accelerometer and gyroscope	(2) $\mu =\frac{1}{N}\sum_{i=1}^{N}Sx_{i}$
Standard deviation	Finds the sensor’s data spread around the mean.	(3) $\sigma =\sqrt{\frac{1}{N}\sum_{i=0}^{N-1}{\left(Sx_{i}-\mu \right)}^{2}}$
Skewness	The measure for the degree of symmetry in the variable distribution.	(4) $Sk_{x}=\frac{\sum_{i=1}^{N}{\left(Sx_{i}-\mu \right)}^{3}}{{\left(\sigma_{x}\right)}^{3}}$
Kurtosis	The measure of tailedness in the variable distribution.	(5) $Kt_{x}=\frac{\sum_{i=1}^{N}{\left(Sx_{i}-\mu \right)}^{4}}{{\left(N\sigma \right)}^{4}}$
Maximum value	Calculates the maximum value of the accelerometer (x,y,z).	(6) $Acc_{max}=maxSx_{i}$
Minimum value	Shows the minimum value of the accelerometer (x,y,z).	(7) $Acc_{min}=minSx_{i}$
Entropy	Essential for differentiating between activities.	(8) $Entropy=-\frac{1}{N}\sum_{i=0}^{N-1}px_{i}logpx_{i}$
Cosine Similarity	To distinguish between activities that fluctuate along an axis.	(9) $\cos\theta =\frac{S_{x}.*S_{y}}{\left|\left|S_{x}\right|\right|\left|\left|S_{y}\right|\right|}$
Root mean square	Calculates the angular movement along the x, y, and z axes, accordingly.	(10) $$RMS_{x}=\sqrt{\frac{1}{N}\sum_{i=1}^{N}Gx_{i}}$$ Where $Gx_{i}$ is sample of *x*-axis gyroscope.
The absolute time difference between peaks	Computed by taking the absolute difference between the maximum and minimum peak times.	(11) $ATD=\left|t_{maxpeak}-t_{minpeak}\right|$
Frequency domain features	To find frequency domain features of acceleration data based on fast Fourier transform (FFT).	(12) $H\left(k\right)=\sum_{n=0}^{N-1}x\left(n\right)e^{-j2\pi (\frac{kn}{N})}$
Quartile Range	To find the middle number between the minimum and the median of the sample data.	(13) $Q_{1}=l+\frac{h}{f}\left(\frac{n}{4}-C\right)$

**Table 5:** Confusion matrix for KNN classifier using Manhattan distance with the cross-validation factor of 10%.

Actual /Predicted	GST-1	GST-2	GST-3	GST-4	GST-5	GST-6	GST-7	GST-8	GST-9	GST-10
**GST-1**	193	2	0	0	1	2	0	0	2	0
**GST-2**	0	194	1	0	1	0	3	1	0	0
**GST-3**	0	0	188	6	2	2	2	0	0	0
**GST-4**	0	2	8	187	0	1	0	2	0	0
**GST-5**	0	2	2	0	176	5	11	2	2	0
**GST-6**	0	1	0	0	2	192	3	2	0	0
**GST-7**	0	0	1	0	11	10	177	1	0	0
**GST-8**	0	0	2	0	7	1	3	187	0	0
**GST-9**	0	0	0	0	2	0	0	0	192	6
**GST-10**	2	0	0	0	0	0	0	0	10	188

**Table 6:** Results for J48 decision tree classifier.

Number of Iterations	Cross-Validations (%)	Accuracy (%)
1	5	86.1
2	10	87.91
3	15	86.6
4	20	87.7
5	25	87.7

**Table 7:** Confusion matrix for J48 classifier with the cross-validation factor of 10%.

Actual/Predicted	GST-1	GST-2	GST-3	GST-4	GST-5	GST-6	GST-7	GST-8	GST-9	GST-10
**GST-1**	191	1	0	1	1	3	0	1	0	2
**GST-2**	0	183	2	6	3	1	4	1	0	0
**GST-3**	0	5	167	9	8	4	1	6	0	0
**GST-4**	0	5	12	167	3	5	2	6	0	0
**GST-5**	0	1	7	2	157	5	20	6	2	0
**GST-6**	3	4	2	7	3	172	8	1	0	0
**GST-7**	0	4	3	1	16	13	162	1	0	0
**GST-8**	2	4	6	7	4	3	5	169	0	0
**GST-9**	0	0	0	0	3	0	0	0	195	2
**GST-10**	3	1	0	0	0	0	0	0	1	195

**Table 8:** Results of RF classifiers with maximum depth and cross-validation factor.

Number of Iterations	Cross Validations (%)	Accuracy (%)
1	5	95.25
2	10	95.9
3	15	95.875
4	20	95.13
5	25	94.25

**Table 9:** Confusion matrix of random forest with accuracy (95.9%).

Actual/Predicted	GST-1	GST-2	GST-3	GST-4	GST-5	GST-6	GST-7	GST-8	GST-9	GST-10
**GST-1**	197	1	0	0	0	0	0	0	2	0
**GST-2**	0	195	0	1	0	0	1	3	0	0
**GST-3**	0	0	191	7	0	1	1	0	0	0
**GST-4**	0	0	4	195	0	1	0	0	0	0
**GST-5**	0	0	5	1	184	2	4	2	2	0
**GST-6**	0	2	1	0	1	194	1	1	0	0
**GST-7**	0	0	0	0	7	7	186	0	0	0
**GST-8**	0	0	2	1	4	1	2	190	0	0
**GST-9**	0	0	0	0	1	0	0	0	194	5
**GST-10**	2	0	0	0	0	0	0	0	6	192

## Data Availability

The data presented in this study are available on request from the corresponding author.
